# Care of the Teeth

**Published:** 1884-08

**Authors:** 


### CARE OF THE TEETH.—DUTY OF DENTISTS.

THERE is, perhaps, as much oversight or neglect by the average dentist, in the matter of cleansing the teeth, in the ordinary cases that come to his care, as in any other particular in practice. How often is it that teeth that have been recently filled will exhibit upon their surfaces more or less of foreign matter, usually salivary calculus? This is sometimes removed from the exposed surfaces, while it is permitted to remain in considerable quantities beneath the margin of the gums.

When the care of a set of teeth and the mouth is committed to the dentist, the first step, so far as treatment and manipulation is concerned, is to render all the teeth thoroughly clean, removing every particle of foreign matter, and polishing the surfaces as perfectly as possible ; giving particular attention to all rough and abraded places. The gums should be rendered healthy and freed from all irritants. In proper and systematic treatment this should precede the operation of filling. Still, in some cases it will be necessary that all go on together, but the rule should be that thorough cleansing precede the operation of filling.

Cleaning the teeth and making the mouth healthy is as important as, and, indeed, more so in some respects than the operation of filling decayed teeth.

A writer in the *Dental Register* says : If the profession could feel the full importance of this, better success would attend the operation of filling.

He who neglects the condition of the mouth in respect to health and purity, and simply fills teeth, irrespective of these conditions, does both himself and patient great injustice. Such operations, however well performed, are far less efficient than they would be if the mouth were kept clean and free from disease. Nor is it enough that the mouth be made healthy and pure, but it must be kept so if the work of the dentist is to be of permanent service. And in order that this good condition of the mouth be maintained the patient should have a clear understanding of its importance and of the means by which it is accomplished, and be made to feel that it is mainly dependent upon himself. It is the duty of the dentist, not only to fully impress this fact upon the mind of his patient, but also to give him all needed information as to the means to be used.

In order that the mouth be kept in pro[illegible]er condi[illegible]ion, it should be examined thoroughly once in from four to twelve months ; with some as often as every four months ; with others once in twelve months will suffice. The dentist who has the be[illegible]t interest of his patients at heart and a just appreciation of his own reputation cannot afford to dismiss them indefinitely, or until the patient finds something breaking down or is admonished by the pain of some active disease.

It is very often that quite faulty fillings in mouths kept healthy and clean seem entirely to arrest decay of the teeth in which they are ; while in mouths that are neglected, impu[illegible]e and diseased, the most perfect fillings utterly fail to save the teeth for any considerable time.

Were dentists as careful in this matter as they ought to be there would be far less of failure in operating upon the natural teeth than is at present realized, and the appreciation of the service of the denthat would be much grea er and his reputation of a higher order than at present, a result to be greatly desired.

